# A Biopsychosocial Profile of Adult Canadians with and without Chronic Back Disorders: A Population-Based Analysis of the 2009-2010 Canadian Community Health Surveys

**DOI:** 10.1155/2014/919621

**Published:** 2014-05-27

**Authors:** Brenna Bath, Catherine Trask, Jesse McCrosky, Josh Lawson

**Affiliations:** ^1^School of Physical Therapy, College of Medicine, University of Saskatchewan, 1121 College Drive, Saskatoon, SK, Canada S7N 0W3; ^2^Centre for Health and Safety in Agriculture, College of Medicine, University of Saskatchewan, 103 Hospital Drive, Saskatoon, SK, Canada S7N 0W8; ^3^Department of Community Health and Epidemiology, College of Medicine, University of Saskatchewan, 103 Hospital Drive, Saskatoon, SK, Canada S7N 0W8

## Abstract

Chronic back disorders (CBD) are a significant public health concern. Profiling Canadians with CBD and the associated biopsychosocial factors at a national population level is important to understand the burden of this condition and how clinicians, health systems, and related policies might address this potentially growing problem. We performed a secondary analysis of the 2009 and 2010 Canadian Community Health Surveys to calculate prevalence and to better understand the differences between people with and without CBD. An estimated 20.2% of the adult Canadian population reports having back problems lasting for 6 months or more. Among people with CBD, there was significantly greater likelihood of living in a more rural or remote location, being Aboriginal, being a former or current smoker, being overweight, having other chronic health conditions, having greater activity limitations, having higher levels of stress, and having lower perceived mental health. People who were single/never married or had an ethnicity other than Caucasian or Aboriginal were less likely to report having CBD. These results contribute to a growing body of research in the area that may assist with strategic prioritization and tailoring of health promotion efforts and health services for people with CBD, particularly among vulnerable groups.

## 1. Introduction

Musculoskeletal disorders comprise a surprisingly large share of the nonfatal disease burden around the world. International data suggest that chronic pain states, such as chronic back disorders (CBD), present a burden at least as great as, or perhaps even greater than, conditions that are conventionally prioritised as public health concerns [1]. In a recent review of the global burden of 290 diseases and conditions, low back pain was found to be the leading cause of years lived with disability [2]. Musculoskeletal conditions, such as low back pain, are likely to increasingly dominate the picture of disability at a population level as the demographic structure of society changes. Low back pain and related disorders have a large societal and personal impact estimated to affect 50–85% of all people at some time in life [3]. The economic consequences of back disorders represent an enormous cost for society due to direct health-care utilization costs and indirect costs such as loss of productivity and lost wages [4]. In Canada, medical expenditures with respect to back disorders are estimated between $6 and $12 billion annually [5]. Although clinical practice guidelines suggest that recovery from an initial onset of acute back pain is usually rapid and complete [6], more recent evidence suggests that persisting (i.e., chronic) pain and disability for up to a year or more are not uncommon [7].

The biopsychosocial model is proposed as a means to more completely understand, evaluate, and manage disability attributed to persisting health conditions such as CBD [8–10]. This model draws on broader understanding of biological and psychosocial influences on the development and persistence of pain and disability; it does not reject a search for important pathology but rather shifts the emphasis to other components of the problem [11]. Even though back pain may start as a physical or biological problem, people with back pain and the health-care providers working with them often view the physical symptom through a series of psychological and social filters [9]. A social model explains disability as being primarily caused by oppressive social and economic conditions. In contrast, a biomedical model has an individual focus and assumes a direct link between pain, disease, and physical pathology. The biopsychosocial approach is a compromise between a purely biomedical and a purely social model of disability and reflects the concept that disability related to CBD should be viewed as a problem arising from the interaction between physical/biological (e.g., age, sex, and physical exposures), psychological (i.e., cognition, affect, and behavior), and social factors (i.e., social and cultural contexts) [8, 9, 12, 13]. The biopsychosocial model forms the basis of the World Health Organization's International Classification of Functioning, Disability, and Health (ICF) [14–16]. The ICF represents a comprehensive classification system that makes it possible to describe disability at a variety of biological, personal, or societal levels in the context of environmental factors that may either enhance or detract from overall health and wellness [14, 16]. The ICF provides a framework in which tissue damage may be a relatively small component of a musculoskeletal problem and acknowledges that psychological, social, and cultural contexts contribute to health outcomes including participation in social and other life activities [17].

A variety of biopsychosocial factors, including age, social support, depression, and other comorbidities, have been linked to persisting pain and disability in people with back disorders [13, 18–20]. In the context of CBD, psychological and social factors are thought to be just as important as (if not more important than) biomedical or physical factors [18, 19, 21]. Prior research using Canadian data has examined predictors of new-onset CBD [22], the association between CBD and depression [20], and self-reported health-care utilization among people with and without CBD [23]. However, to the best of our knowledge, investigation of both the prevalence of CBD and examination of the association of a range of biopsychosocial factors with CBD in a population-based sample of Canadian adults has yet to be done. Notably, much of the population-based back pain research internationally has focused on prevalence of back pain (using a range of case definitions) [24–26] or focused on a narrow subset of biopsychosocial factors, such as age, gender, depression, or occupational risk factors [24, 26–28]. Furthermore, there appears to be very few countrywide studies on CBD prevalence, particularly ones that consider a range of biopsychosocial factors [24]. Among 165 included studies in a systematic review of the global prevalence of low back pain, only 13 (7.9%) examined chronic (i.e., >3-month duration) or recurrent low back pain, and among those, only 2 studies had a nationally representative sample [20, 29]. However, neither nationally representative study examined CBD alone as the dependent variable of interest or profiled the biological, psychological, and social factors associated with CBD. Currie and colleagues focused on the relationship between CBD and depression [20]. Makela et al. investigated how a range of musculoskeletal disorders (including CBD) were associated with disability, but not the biopsychosocial factors associated with CBD [29].

Determining the prevalence of CBD and profiling its associated biological, psychological, and social factors at a national population level are important to understand the burden of this condition and how clinicians, health systems, and related policies might address this potentially growing public health problem. The aim of this study was to determine the prevalence of self-reported CBD and to profile the sociodemographics, comorbidities, perceived disability, and other health status indicators among people with CBD compared to people without CBD in the Canadian adult population.

## 2. Materials and Methods 

### 2.1. Study Design and Data Source

We used data from Statistics Canada's 2009 and 2010 Canadian Community Health Surveys (CCHS). The CCHS was designed to provide a flexible survey instrument to address emerging health issues in Canada. It includes a range of content such as sociodemographics, health status, health behaviours, and many other determinants of health [30]. The CCHS is a cross-sectional study in which respondents are selected using a complex survey design with a two-phase stratified sampling plan intended to ensure adequate representation from each Canadian region.

### 2.2. Study Population

The CCHS targets Canadians aged 12 years and older living in private dwellings in all 10 provinces and 3 territories. The survey did not include people living on First Nations reserves or residents of institutional and some noninstitutional collectives (e.g., military bases, Canadian Armed Forces vessels, merchant and coast guard vessels, campgrounds, or parks). Approximately 130 000 Canadians were selected for the 2009 or 2010 survey, sampled from and representative of approximately 98% of the Canadian population aged 12 years and older. The participation rate of this survey was 72.3% [30]. The focus of our analysis was persons aged 18 years and older (*N* = 113 647). Of these adult respondents, 25 545 reported having a CBD.

Ethical approval for data collection was completed by Statistics Canada (Government of Canada). Our access to this data was only through approved research data centers following a rigorous screening process and approval of the proposed research in order to use this deidentified data as well as vetting procedures to ensure confidentiality and protection of the subject.

### 2.3. Survey Data and Operational Definitions

The dependent variable was presence of CBD, using the survey question: “(Do you) have back problems, excluding fibromyalgia and arthritis?” This section of the survey is prefaced with the reminder: “Now I'd like to ask about certain chronic health conditions which (you) may have. We are interested in ‘long-term conditions' which are expected to last or have already lasted for 6 months or more and that have been diagnosed by a health professional.”

A range of independent variables grouped into sociodemographic, lifestyle, and health characteristics were identified based on a review of the literature, clinical relevance, and data availability within the survey. The alignment of these variables with the biopsychosocial model was a further consideration for inclusion in the study. Further details regarding the description and categorization of the variables can be found in Table 1.

### 2.4. Statistical Analysis

The descriptive analysis included calculation of proportions over each of our independent variables (all categorical) for persons with and without CBD using a chi-squared test suitable for complex survey data to test whether each variable was distributed differently between those with and without CBD. Crude associations between each independent variable and CBD were further assessed using bivariate logistic regression. The strength of association was quantified with the odds ratio (OR) and 95% confidence interval (CI).

In order to control for potential confounding, a multiple logistic regression model was developed. Due to the associations between many of our ordinal independent variables, a Goodman and Kruskal gamma was calculated between each pair of independent variables to determine their degrees of association. The regression model was then fitted using purposeful selection informed by statistical significance, clinical importance, potential and observed confounding effects, and the calculated gamma values. We also considered interaction terms: sex by age and sex by Health Utility Index (HUI) pain and function index. Of the interaction terms, only sex by age was retained.

All analyses were performed using Stata 13 software with built-in survey data tools for probability weights and bootstrapping. Probability weights provided by Statistics Canada were used to account for unequal probability of selection, and bootstrap methods for robust variance estimation were employed using bootstrap weights provided by Statistics Canada in order to account for the complex survey design and to accurately estimate standard errors.

## 3. Results

Estimated CBD prevalence of 20.2% in the adult Canadian population was observed.  Table 2 presents the results of our descriptive and bivariate analysis. When comparing adults with and without CBD, all of the sociodemographic, lifestyle, and health characteristics we examined were significantly different between these groups at the *P* < 0.05 level. Among those with CBD, a higher proportion were female compared to those who did not have CBD. Respondents with higher levels of educational attainment and higher income levels (i.e., higher income adequacy quintile) were less likely to report having CBD. People living in more rural and remote regions (i.e., moderate, weak, or no metropolitan influence zones (MIZ)) were more likely than urban or strongly influenced MIZ dwellers to report having CBD. Aboriginal respondents were more likely than Caucasians to report CBD, whereas people with “other” ethnicity were less likely than Caucasians to report CBD.

The top 5 self-reported chronic comorbidities among people with CBD were arthritis, high blood pressure, migraines, asthma, and mood disorders. Respondents who reported having any of these other chronic conditions were more likely to report having CBD. Also, people reporting having 1-2 or 3 or more chronic health conditions (other than CBD) were more likely to report having CBD. Lower physical activity levels and higher time spent being sedentary were associated with a greater likelihood of reporting CBD. People with CBD were more likely than those without CBD to report having activity limitations due to pain.

**Figure 1:**
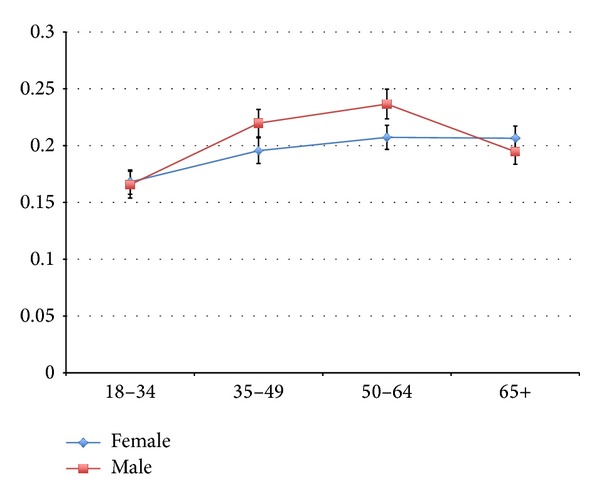
Predicted probabilities of CBD by age and sex.

Table 3 presents our multivariate model along with the unadjusted (bivariate) logistic regression results for each variable included in the model. All variables except residence in a strongly influenced MIZ were significant in the bivariate analysis. In the adjusted model, all variables were found to be significant except sex, education (all levels), residence in strongly or moderately influenced MIZ, being obese, and physical activity. Due to an observed interaction between sex and age category, we present a graph of predicted probability of CBD over age for each sex in Figure 1.

## 4. Discussion

The aim of this study was to determine the prevalence of CBD and to profile a range of variables, framed by a biopsychosocial model, among people with CBD compared to people without CBD in the Canadian adult population. Profiling those with CBD and its associated biological, psychological, and social factors at a national population level is important to understand the burden of this condition and how clinicians, health systems, and related policies might address this potentially growing public health problem.

We found that a substantial proportion of adult Canadians reported having back problems lasting for 6 months or more. Women had higher prevalence of CBD than men overall, but the relationship depended on age. The prevalence of CBD among men in our sample followed the typical clinical pattern described in the literature, whereby prevalence is highest in middle-aged groups (e.g., 50–64 years) and tapers off with increasing age [22]. However, this pattern was not evident among women with the prevalence of CBD among women aged 65 or more being similar to that among women aged 50–64 years. Among people with CBD, there was significantly greater likelihood of living in a more rural or remote location, being Aboriginal, being a former or current smoker, being overweight, having other chronic health conditions, having increased levels of perceived pain and activity limitations, having higher levels of stress, and having lower perceived mental health. People who were single/never married or had an ethnicity other than Caucasian or Aboriginal were less likely to report having CBD.

Comparison of studies on prevalence of back disorders is challenging due to heterogeneity across research methods, case definitions, and study populations [26]. Back disorders include a large heterogeneous group of clinical and etiological entities [35]. The most common descriptor used in epidemiological studies is “low back pain” which can represent a variety of underlying clinical conditions and duration of symptoms. Given these issues, it is unsurprising that population-based estimates of low back pain prevalence vary substantially worldwide. An estimated 15% to 20% of adults experience back pain during a single year, and 50% to 80% experience at least one episode of back pain during their lifetime [3, 24]. The sex differences we found in our study are similar to those of a recent systematic review, which found that prevalence of low back pain was higher in women overall and among older women [24]. Even though other research has shown that women are more likely to develop chronic low back pain and have higher perceived disability due to back pain [36], we did not find that sex was associated with perceived activity limitations due to pain (i.e., HUI pain and function). However, a large proportion of people with CBD reported having some degree of activity limitations, with 10.0% reporting “most” activities were limited due to pain. This finding is similar to another study in which 10.5% of an adult Australian sample reported experiencing high disability due to low back pain [25]. Further to this, a Canadian study found that musculoskeletal conditions in general were the most prevalent medical condition (46.1%) to which activity and participation limitations were attributed [37].

There are a number of environmental, personal, and lifestyle factors that could potentially influence the onset and course of back pain. An inverse relationship between social status and educational attainment with the occurrence of back pain has been well documented in prior research [26] and confirmed in our study. Similar to our findings, Zvolensky et al. found that people with CBD were more likely to smoke than those without, an association that remained significant after adjusting for a variety of sociodemographic factors and the presence of mood or anxiety disorders [38]. The relationship between CBD and being overweight or obese and physical activity levels is equivocal as yet [39, 40], a finding echoed by our results. Psychological factors such as depression have been shown to be associated with having CBD and the development of chronic back pain [20, 41]. Although “depression” was not included as a variable in our final adjusted model, self-rated mental health and perceived stress were. Further to depression, there are a number of other comorbidities that we found to be associated with CBD. A systematic review of comorbidities and low back pain found a positive association between a number of other disorders (e.g., migraines/headaches, respiratory, and cardiovascular conditions); however, the nature of the relationship between these comorbidities and CBD is unclear [42]. Not only the presence of certain chronic conditions but also the number of other conditions was highly associated with having CBD. Thus, the issue of multimorbidity is likely an important consideration when examining health-care policies and services related to CBD.

To the best of our knowledge, the higher CBD prevalence in rural and Aboriginal Canadian populations has not previously been reported; however, these findings are not surprising given the well-documented health disparities in these populations [43]. Although higher rates of arthritis are documented in Aboriginal Canadians [43, 44], no studies have focused solely on CBD. The higher prevalence of CBD among Aboriginal people and rural and remote residents calls for further investigation into whether these groups have different biopsychosocial characteristics and thus perhaps different needs in terms of health care or health promotion related to CBD. Research examining CBD among Aboriginal people in Australia, for example, found that the condition can be profoundly disabling and that issues of sex, cultural obligations, and emotional consequences are important considerations for health care [45].

The results of this study should be considered in light of a number of limitations. The cross-sectional design does not permit conclusions regarding the direction of the observed associations nor does it capture the clinical course and lifetime progression of CBD. However, the national population cross-sectional approach we used in this study does allow us to capture not only new-onset or incident CBD but also those people with existing and potentially longstanding CBD. The classification of having CBD was derived through self-report and may have been variably interpreted by respondents. Back disorders include a large group of clinical and etiological entities and there is no “gold standard” for clinical classification and diagnosis for many of these conditions. The International Classification of Diseases- (ICD-) 10 system does not have an adequate and distinct diagnostic code for chronic pain or CBD [1]. The development and international acceptance of a standard case definition for CBD would help to move the research and policy agenda for CBD forward. Although we attempted to examine a range of independent variables guided by the biopsychosocial model and identified based on a review of the literature, clinical relevance, and data availability within the survey, not all relevant variables may have been considered. Specifically, this study did not examine health-care utilization or other economic impact factors. However, many people with CBD may not necessarily seek health care for their condition or seek care during the time frame specified by the survey. In addition, CCHS data are only available for Aboriginal people living off of reserves and this analysis is limited to the categories “Aboriginal,” “Caucasian,” and “other” which are not homogeneous groups. Finally, the self-reported data on which this study is based may underestimate the prevalence of some behavioural risk factors, such as overweight, obesity, and smoking and overestimate the prevalence of physical activity [46].

## 5. Conclusion

This study provides insight into the magnitude and nature of CBD in Canada using a nationally representative sample. We found that a substantial proportion (20.2%) of adult Canadians report having back problems lasting for 6 months or more. A variety of modifiable and nonmodifiable sociodemographic, health, and lifestyle factors were significantly associated with having CBD. Our results demonstrated that some biological, psychological, and sociodemographic factors are more common among Canadians with CBD than those without. Regardless of causality, understanding the unique characteristics of people with CBD can help develop more appropriate educational materials or programs (for prevention or management) and help clinicians consider potential additional health needs or potential management contraindications presented by comorbidities. Consideration of factors such as rural and remote residence, Aboriginal ethnicity, and multimorbidity may have implications for ensuring equitable access to appropriate health services and health promotion efforts. Further research should examine CBD in these potentially vulnerable groups and examine issues of health-care utilization, access, and unmet health needs.

## Figures and Tables

**Table 1:** Description of (independent) variables included in analysis.

Variable	Description (if applicable) and categories
Sociodemographic characteristics	
Age	18–34 yrs; 35–49 yrs; 50–64 yrs; ≥65 yrs. Categories based on quartiles and clinical relevance
Sex	Male; female
Education	Less than secondary; secondary graduation; some postsecondary; postsecondary graduation
Income	A StatsCan-derived variable addressing income adequacy. Quintile of adjusted ratio of total household income to the low income cut-off corresponding to household and community size. This variable was unavailable for some respondents, for example, in cases where the person most knowledgeable about the household could not be identified (*N* = 92,669)
Residence	A StatsCan-derived variable. “Urban” residence includes communities with populations ≥10,000 people. “Rural” communities are disaggregated into subgroups or metropolitan influenced zones (MIZ) based on the size of commuting flows to any larger urban center [31]
Ethnicity	Caucasian; Aboriginal (i.e., North American Indian, Métis, or Inuit); other
Marital status	Single; married or common law; widowed or separated or divorced
Body Mass Index (BMI)	Derived from self-reported height and weight Underweight and normal (<25 kg/m^2^); overweight (25–29.9 kg/m^2^); obese (≥30 kg/m^2^) [32]

Lifestyle characteristics	
Smoking status	Never smoked; former smoker; current smoker
Physical activity-transportation and leisure	A StatsCan-derived variable combining leisure time and transportation-physical activity based on estimated total daily energy expenditure variables (kcal/kg/day): active; moderately active; inactive
Sedentary activity duration	A StatsCan-derived variable of total number of hours per week spent in sedentary activities (excluding reading): 0–14 hours; 15–24 hours; 25–39 hours; 40 or more hours.* This variable was only available for respondents in the provinces of Newfoundland, Manitoba, and British Columbia (*N* = 22,380)

Health characteristics	
Number of other comorbidities/chronic conditions	Includes “long-term conditions” which are expected to last or have already lasted for 6 months or more and that have been diagnosed by a health professional. No other chronic conditions (other than CBD); 1 or 2 chronic conditions (other than CBD); 3 or more chronic conditions (other than CBD)
Type of other comorbidities	Presence of top 5 chronic comorbidities associated with CBD: arthritis (excluding fibromyalgia); high blood pressure; migraine headaches; asthma; mood disorders (i.e., depression, bipolar disorder, mania, or dysthymia)
Perceived disability	This variable, derived from the Health Utility Index (HUI) [33], considers whether pain prevents a person from performing activities of daily living. 5 categories: no pain or discomfort; pain prevents no activities; pain prevents a few activities; pain prevents some activities; pain prevents most activities
Depression probability	A StatsCan-derived variable indicating the probability that the respondent would have been diagnosed as having experienced a major depressive episode in the past 12 months, if they had completed the long-form composite international diagnostic interview (CIDI) [34]. This variable was not available for respondents that completed the survey by proxy (*N* = 53,017)
Self-rated stress	Ability to handle day-to-day demands: not at all/not very; a bit; quite a bit/extremely*
Self-rated mental health	Indicating the respondent's mental health status based on his/her own judgement: excellent/very good; good; fair/poor*
Self-rated overall health	Indicating the respondent's health status based on his/her own judgement or his/her proxy: excellent/very good; good; fair/poor*
Self-rated work stress	Indicating level of stress encountered “most days at work”: not at all/not very; a bit; quite a bit/extremely.* This variable was only available for employed respondents (*N* = 69,992)

*Collapsing of these categories was performed to maintain equal-sized categories and consistent categorization for all variables of interest.

**Table 2:** Sociodemographic and lifestyle characteristics of adult Canadians with and without CBD.

	Proportions			Unadjusted odds ratio	
	With CBD	Without CBD	*P* value (chi-square)	OR (95% CI)	*P* value
Age			<0.001		
18–34	**16.7%**	**31.8%**		Reference category	
35–49	**28.0%**	**28.6%**		**1.87 (1.72–2.02)**	<0.001
50–64	**33.1%**	**24.0%**		**2.63 (2.44–2.83)**	<0.001
65+	**22.3%**	**15.6%**		**2.71 (2.53–2.91)**	<0.001
Sex	0.005	
Female	**52.2%**	**50.5%**		Reference category	
Male	**47.8%**	**49.5%**		**0.93 (0.88–0.98)**	0.005
Education	<0.001	
Less than secondary	**19.2%**	**13.6%**		Reference category	
Secondary graduation	**16.5%**	**17.2%**		**0.68 (0.63–0.74)**	<0.001
Some postsecondary	**7.4%**	**8.5%**		**0.62 (0.56–0.70)**	<0.001
Postsecondary graduation	**56.9%**	**60.7%**		**0.66 (0.62–0.71)**	<0.001
Income quintile	<0.001	
1	**24.2%**	**18.6%**		Reference category	
2	**21.3%**	**19.7%**		**0.83 (0.76–0.90)**	<0.001
3	**18.9%**	**20.2%**		**0.72 (0.66–0.78)**	<0.001
4	**17.2%**	**20.7%**		**0.63 (0.58–0.69)**	<0.001
5	**18.4%**	**20.8%**		**0.68 (0.62–0.74)**	<0.001
MIZ	<0.001	
Urban/metropolitan	**81.1%**	**83.8%**		Reference category	
Rural strongly influenced	**3.8%**	**3.9%**		1.02 (0.91–1.14)	0.749
Rural moderately influenced	**7.6%**	**6.2%**		**1.26 (1.16–1.37)**	<0.001
Rural weak/uninfluenced + territories	**7.5%**	**6.1%**		**1.28 (1.20–1.37)**	<0.001
Ethnicity	<0.001	
Caucasian	**84.0%**	**79.9%**		Reference category	
Aboriginal	**4.2%**	**2.7%**		**1.46 (1.30–1.65)**	<0.001
Other	**11.8%**	**17.3%**		**0.64 (0.58–0.71)**	<0.001
Marital status	<0.001	
Single	**16.3%**	**24.6%**		Reference category	
Married + common law	**65.7%**	**63.3%**		**1.57 (1.47–1.67)**	<0.001
Widowed + separated + divorced	**18.0%**	**12.0%**		**2.27 (2.09–2.46)**	<0.001
Smoking status	<0.001	
Never smoked	**30.7%**	**40.4%**		Reference category	
Former smoker	**42.8%**	**39.1%**		**1.44 (1.35–1.53)**	<0.001
Current smoker	**26.5%**	**20.5%**		**1.70 (1.58–1.82)**	<0.001
BMI			<0.001	
Underweight/normal	**40.1%**	**50.1%**		Reference category	
Overweight	**36.8%**	**33.2%**		**1.38 (1.30–1.47)**	<0.001
Obese	**23.1%**	**16.7%**		**1.72 (1.61–1.84)**	<0.001
Number of comorbidities	<0.001	
None	**27.5%**	**55.9%**		Reference category	
1-2	**46.7%**	**36.3%**		**2.62 (2.46–2.79)**	<0.001
3+	**25.8%**	**7.9%**		**6.67 (6.20–7.16)**	<0.001
Arthritis	<0.001	
No	**63.4%**	**88.6%**		Reference category	
Yes	**36.6%**	**11.4%**		**4.48 (4.24–4.74)**	<0.001
High BP	<0.001	
No	**73.6%**	**83.4%**		Reference category	
Yes	**26.4%**	**16.6%**		**1.80 (1.70–1.91)**	<0.001
Migraines	<0.001	
No	**81.7%**	**91.7%**		Reference category	
Yes	**18.3%**	**8.3%**		**2.47 (2.30–2.66)**	<0.001
Asthma	<0.001	
No	**87.3%**	**93.2%**		Reference category	
Yes	**12.7%**	**6.8%**		**2.01 (1.85–2.19)**	<0.001
Mood disorders	<0.001		
No	**86.9%**	**94.7%**		Reference category	
Yes	**13.1%**	**5.3%**		**2.73 (2.52–2.95)**	<0.001
Physical activity-transportation and leisure	<0.001		
Active	**22.3%**	**28.0%**		Reference category	
Moderately active	**23.3%**	**26.2%**		**1.11 (1.04–1.21)**	0.008
Inactive	**54.3%**	**45.8%**		**1.48 (1.39–1.58)**	<0.001
Sedentary activity	<0.001		
0 to 14 hours	**21.9%**	**25.2%**		Reference category	
15 to 24 hours	**29.2%**	**31.6%**		1.06 (0.91–1.25)	0.433
25 to 39 hours	**29.6%**	**27.4%**		**1.24 (1.07–1.45)**	0.005
40 or more hours	**19.3%**	**15.9%**		**1.40 (1.18–1.66)**	<0.001
Perceived disability	<0.001	
No pain or discomfort	**53.5%**	**88.7%**		Reference category	
Pain prevents no activities	**9.5%**	**3.8%**		**4.12 (3.73–4.55)**	<0.001
Pain prevents a few activities	**14.2%**	**3.7%**		**6.40 (5.84–7.01)**	<0.001
Pain prevents some activities	**12.7%**	**2.3%**		**9.03 (8.17–9.98)**	<0.001
Pain prevents most activities	**10.0%**	**1.5%**		**11.32 (9.99–12.82)**	<0.001
Depression scale predicted probability			<0.001	
<0.9	**90.5%**	**95.5%**		Reference category	
≥0.9	**9.5%**	**4.5%**		**2.24 (1.97–2.56)**	<0.001
Self-rated stress	<0.001		
Not at all/not very	**28.0%**	**35.9%**		Reference category	
A bit	**41.0%**	**42.2%**		**1.25 (1.18–1.32)**	<0.001
Quite a bit/extremely	**31.1%**	**21.9%**		**1.83 (1.71–1.95)**	<0.001
Self-rated mental health	<0.001		
Excellent/very good	**62.2%**	**76.5%**		Reference category	
Good	**27.4%**	**19.4%**		**1.74 (1.64–1.85)**	<0.001
Fair/poor	**10.3%**	**4.1%**		**3.08 (2.82–3.38)**	<0.001
Self-rated overall health	<0.001		
Excellent/very good	**41.1%**	**64.1%**		Reference category	
Good	**34.1%**	**27.1%**		**1.96 (1.85–2.08)**	<0.001
Fair/poor	**24.8%**	**8.7%**		**4.43 (4.12–4.75)**	<0.001
Self-rated work stress	<0.001		
Not at all/not very	**22.8%**	**29.0%**		Reference category	
A bit	**39.2%**	**42.2%**		**1.18 (1.08–1.29)**	<0.001
Quite a bit/extremely	**38.1%**	**28.8%**		**1.68 (1.53–1.84)**	<0.001

MIZ: metropolitan influenced zone; BMI: Body Mass Index; BP: blood pressure.

**Table 3:** Multivariate model of adult Canadians with and without CBD.

	Odds ratio for CBD			
	Unadjusted		Adjusted	
	OR (95% CI)	*P* value	OR (95% CI)	*P* value
Age				
18–34	Reference category		Reference category	
35–49	**1.87 (1.73–2.01)**	<0.001	**1.25 (1.12–1.4)**	<0.001
50–64	**2.63 (2.45–2.82)**	<0.001	**1.36 (1.21–1.53)**	<0.001
65+	**2.71 (2.54–2.9)**	<0.001	**1.35 (1.2–1.53)**	<0.001
Sex		
Female	Reference category		Reference category	
Male	**0.93 (0.89–0.98)**	0.005	0.98 (0.87–1.1)	0.739
Age ∗ male			
18–34			Reference category	
35–49		**1.22 (1.04–1.42)**	0.012
50–64		**1.25 (1.06–1.48)**	0.009
65+		0.93 (0.8–1.09)	0.384
Education		
Less than secondary	Reference category		Reference category	
Secondary graduation	**0.68 (0.63–0.74)**	<0.001	0.99 (0.89–1.09)	0.803
Some postsecondary	**0.62 (0.56–0.69)**	<0.001	0.93 (0.82–1.06)	0.267
Postsecondary graduation	**0.66 (0.62–0.71)**	<0.001	1.01 (0.93–1.09)	0.819
MIZ		
Urban/metropolitan	Reference category		Reference category	
Rural strongly influenced	1.02 (0.91–1.15)	0.749	0.95 (0.83–1.08)	0.446
Rural moderately influenced	**1.26 (1.17–1.36)**	<0.001	1.08 (0.98–1.19)	0.102
Rural weak/uninfluenced + territories	**1.28 (1.19–1.38)**	<0.001	**1.09 (1–1.18)**	0.046
Ethnicity		
Caucasian	Reference category		Reference category	
Aboriginal	**1.46 (1.3–1.65)**	<0.001	**1.23 (1.06–1.43)**	0.007
Other	**0.64 (0.58–0.71)**	<0.001	**0.84 (0.76–0.94)**	0.002
Marital status		
Single	Reference category		Reference category	
Married + common law	**1.57 (1.47–1.67)**	<0.001	**1.15 (1.06–1.25)**	0.001
Widowed + separated + divorced	**2.27 (2.09–2.46)**	<0.001	**1.17 (1.05–1.31)**	0.004
Smoking status		
Never smoked	Reference category		Reference category	
Former smoker	**1.44 (1.36–1.53)**	<0.001	**1.15 (1.07–1.23)**	<0.001
Current smoker	**1.7 (1.58–1.81)**	<0.001	**1.39 (1.29–1.51)**	<0.001
BMI		
Underweight/normal	Reference category		Reference category	
Overweight	**1.38 (1.31–1.47)**	<0.001	**1.11 (1.04–1.19)**	0.001
Obese	**1.72 (1.61–1.83)**	<0.001	1.02 (0.95–1.1)	0.576
Number of comorbidities		
None	Reference category		Reference category	
1-2	**2.62 (2.47–2.78)**	<0.001	**1.78 (1.67–1.91)**	<0.001
3+	**6.67 (6.21–7.16)**	<0.001	**2.68 (2.42–2.97)**	<0.001
Physical activity-transportation + leisure				
Active	Reference category		Reference category	
Moderately active	**1.11 (1.03–1.2)**	0.008	0.94 (0.87–1.03)	0.19
Inactive	**1.48 (1.39–1.58)**	<0.001	0.98 (0.92–1.06)	0.677
Perceived disability			
No pain or discomfort	Reference category		Reference category	
Pain prevents no activities	**4.12 (3.74–4.54)**	<0.001	**3.21 (2.89–3.56)**	<0.001
Pain prevents a few activities	**6.4 (5.87–6.98)**	<0.001	**4.67 (4.22–5.17)**	<0.001
Pain prevents some activities	**9.03 (8.12–10.03)**	<0.001	**5.84 (5.19–6.56)**	<0.001
Pain prevents most activities	**11.32 (10.12–12.65)**	<0.001	**6.35 (5.56–7.25)**	<0.001
Self-rated stress				
Not at all/not very	Reference category		Reference category	
A bit	**1.25 (1.17–1.33)**	<0.001	**1.18 (1.1–1.27)**	<0.001
Quite a bit/extremely	**1.83 (1.71–1.95)**	<0.001	**1.33 (1.23–1.45)**	<0.001
Self-rated mental health				
Excellent/very good	Reference category		Reference category	
Good	**1.74 (1.64–1.85)**	<0.001	**1.22 (1.14–1.32)**	<0.001
Fair/poor	**3.08 (2.82–3.37)**	<0.001	**1.29 (1.14–1.45)**	<0.001

MIZ: metropolitan influenced zone; BMI: Body Mass Index; BP: blood pressure.
